# Association between vector-borne pathogen seroprevalence in shelter-housed and owned dog populations in the contiguous United States of America

**DOI:** 10.1186/s13071-023-05994-9

**Published:** 2023-11-07

**Authors:** Jenna R. Gettings, Christopher S. McMahan, Christopher A. Cleveland, Andrea Varela-Stokes, Kris Hubbard, Sarah A. Hamer, Heather S. Walden, Michael J. Yabsley

**Affiliations:** 1 Southeastern Cooperative Wildlife Disease Study, Department of Population Health, College of Veterinary Medicine, University of Georgia, Athens, Georgia 30602 USA; 2 School of Mathematical and Statistical Sciences, Clemson University, Clemson, South Carolina 29634 USA; 3 Department of Comparative Biomedical Sciences, College of Veterinary Medicine, Mississippi State University, Starkville, Mississippi 39762 USA; 4 Department of Pathobiology and Population Medicine, Mississippi State University College of Veterinary Medicine, Mississippi, 39762 USA; 5 Department of Veterinary Integrative Biosciences, School of Veterinary Medicine & Biomedical Sciences, Texas A&M University, College Station, Texas 77843 USA; 6 Department of Comparative, Diagnostic and Population Medicine, College of Veterinary Medicine, University of Florida, Gainesville, Florida 32608 USA; 7 Warnell School of Forestry and Natural Resources, University of Georgia, Athens, Georgia 30602 USA; 8 Center for the Ecology of Infectious Diseases, University of Georgia, University of Georgia, Athens, Georgia 30602 USA; 9 Department of Comparative Pathobiology, Cummings School of Veterinary Medicine, Tufts University, North Grafton, MA 01536 USA

**Keywords:** Canine, Ticks, Tick-borne diseases, Zoonoses

## Abstract

Domestic dogs are susceptible to numerous vector-borne pathogens that are of significant importance for their health. In addition to being of veterinary importance, many of these pathogens are zoonotic and thus may pose a risk to human health. In the USA, owned dogs are commonly screened for exposure to or infection with several canine vector-borne pathogens. Although the screening data are widely available to show areas where infections are being diagnosed, testing of owned dogs is expected to underestimate the actual prevalence in dogs that have no access to veterinary care. The goal of this study was to measure the association between the widely available data from a perceived low-risk population with temporally and spatially collected data from shelter-housed dog populations. These data were then used to extrapolate the prevalence in dogs that generally lack veterinary care. The focus pathogens included *Dirofilaria immitis*, *Ehrlichia* spp., *Anaplasma* spp., and *Borrelia burgdorferi.* There was a linear association between the prevalence of selected vector-borne pathogens in shelter-housed and owned dog populations and, generally, the data suggested that prevalence of heartworm (*D. immitis*) infection and seroprevalence of *Ehrlichia* spp. and *B. burgdorferi* are higher in shelter-housed dogs, regardless of their location, compared with the owned population. The seroprevalence of *Anaplasma* spp. was predicted to be higher in areas that have very low to low seroprevalence, but unexpectedly, in areas of higher seroprevalence within the owned population, the seroprevalence was expected to be lower in the shelter-housed dog population. If shelters and veterinarians make decisions to not screen dogs based on the known seroprevalence of the owned group, they are likely underestimating the risk of exposure. This is especially true for heartworm. With this new estimate of the seroprevalence in shelter-housed dogs throughout the USA, shelters and veterinarians can make evidence-based informed decisions on whether testing and screening for these pathogens is appropriate for their local dog population. This work represents an important step in understanding the relationships in the seroprevalences of vector-borne pathogens between shelter-housed and owned dogs, and provides valuable data on the risk of vector-borne diseases in dogs.

**Graphical abstract:**

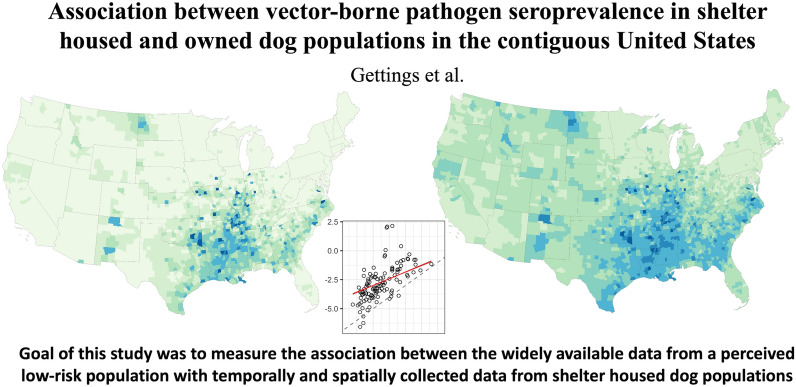

**Supplementary Information:**

The online version contains supplementary material available at 10.1186/s13071-023-05994-9.

## Background

Domestic dogs are susceptible to numerous vector-borne pathogens that are of significant importance for their health [1, 2]. In addition to being of veterinary importance, many of these pathogens are zoonotic and thus may pose a risk to human health [1, 2]. In the USA, dogs are commonly screened for exposure to or infection with several canine vector-borne pathogens, including *Dirofilaria immitis*, *Ehrlichia canis*, *Ehrlichia ewingii*, *Anaplasma platys*, *Anaplasma phagocytophilum*, and *Borrelia burgdorferi* [1–4]. The distribution and prevalence of these pathogens can be estimated from the screening data, allowing veterinarians and the public to determine the risk of exposure for dogs [2–4]. However, one limitation of these data is that they are collected primarily from owned dogs under the care of a veterinarian. Dogs not under the care of a veterinarian or free-roaming dogs are less likely to be tested or have their results included in these datasets. These dogs are assumed to be at a higher risk of exposure to vector-borne pathogens because many chemoprophylactic products used against flea, tick, and mosquito vectors or pathogens, are prescription based. Owners that do not regularly visit a veterinarian with their dog may be unaware of the risks to their pet and the products available to protect them. Additionally, free-roaming dogs are less likely to have shelter from vectors. As a result, the prevalence of vector-borne pathogens within this population is expected to be higher compared to those under the consistent care of a veterinarian [5].

Higher risk populations of dogs (e.g., those in shelters or displaced by natural disasters) are hypothesized to have high prevalences of vector-borne pathogens, and the few studies that have directly compared the seroprevalence of higher risk dog populations to the seroprevalence of a lower risk population (e.g., owned and under the care of a veterinarian) support this hypothesis [6–14]. However, these studies were limited in scope and geographic distribution of data, which limits our ability to explore the relationship between the prevalences of vector-borne pathogens in these two groups of dogs.

The goal of this study was to measure the association between the widely available data from a wide geographical area of a perceived low-risk population and comparable data from select shelter-housed dog populations. In the future, this association can then be used to better estimate the rate of pathogen exposure for dogs in areas that have not been sampled. Ultimately, we aim to improve our understanding of the risk of exposure to these pathogens in a general population of dogs, not just those under the care of a veterinarian.

## Methods

### Classification of study groups

For the purposes of this study, we classified the general canine population (i.e., all dogs within the USA) into two groups based on their expected level of risk for any of the four considered pathogens (*D. immitis*, *Ehrlichia* spp., *Anaplasma* spp., and *B. burgdorferi*). Individually owned dogs receiving routine veterinary care may be more likely to receive prophylaxis and increased protection against exposure, and thus are at lower risk of infection. Shelter-housed dogs represent a canine population that is less likely to have a history of regular veterinary care and provision of prophylaxis, while having decreased protection against physical exposure to a vector (i.e., they are predominately outside dogs). Based on these descriptions, we used two datasets for this analysis. The first dataset is from the Companion Animal Parasite Council (CAPC; (www.capcvet.org), for which the data have been compiled from two veterinary diagnostic laboratories since 2011. Most of these dogs are under the care of a veterinarian, and are therefore assumed to be at lower risk of exposure; they are henceforth referred to as the “owned dog population.” The second is a prospective dataset of samples from shelter-housed dogs representing the high-risk population [14]. Shelter-housed dogs likely represent a mixed population in terms of the level of risk of exposure, but they are much more accessible compared to stray or feral dogs, or owned dogs that are not under the care of a veterinarian.

### Data

#### Owned dog population

For this study, data from Idexx Laboratories (Westbrook, ME) included results from SNAP® 4Dx Plus® tests performed at reference laboratories and veterinary clinics using the SNAPshot Dx® or SNAP Pro analyzer, which allow the transfer of data to a centralized database. The SNAP test is a point-of-care/in-clinic enzyme-linked immunosorbent assay that detects antigen from *D. immitis* (heartworm) and antibodies against *B. burgdorferi*, *Anaplasma* spp., and *Ehrlichia* spp. [15]. Data from Antech Diagnostics (Fountain Valley, CA) included the results of *D. immitis* antigen (Dirochek®) well-based enzyme-linked immunosorbent assays performed within reference laboratories [16]. Both Idexx Laboratories and Antech Diagnostics provide monthly aggregated count data to the CAPC on a county scale. From these data, the seroprevalence of these vector-borne infections could be calculated. For this study, data from January 2013 up to and including December 2019 were aggregated by county. Over 67 million test results were available for heartworm and approximately 32 million for *B. burgdorferi*, *Ehrlichia* spp., and *Anaplasma* spp.; the overall prevalence for each was 1.3%, 5.92%, 2.91%, and 3.27%, respectively. Data are available from approximately two-thirds of the 3106 counties in the contiguous USA, with an average of 10,000–20,000 tests per county. These counts (total positive and total tests performed) were used in the spatial convolution model described below to interpolate estimates of seroprevalence for counties that were missing data.

#### Shelter-housed population

Data from shelters were obtained from multiple sources. First, Idexx Laboratories provided a subset of the data described above that included test results submitted by shelters between January 2017 and September 2017. These data were more likely to be found for large metropolitan areas and many were from areas with low seroprevalence of the pathogens of interest. To broaden the representativeness of the data, we recruited shelters representing 26 counties in areas targeted because of a known high seroprevalence of vector-borne exposure [1, 2, 4, 14]. Recruitment was performed either by directly contacting shelters or through the distribution of recruitment letters by state veterinary medical associations. Participating shelters were asked to test all dogs that entered the shelter except those less than 6 months of age, under a bite quarantine, or known to have been relocated from outside the county or neighboring counties. The SNAP® 4Dx Plus tests were provided by Idexx Laboratories and shipped directly to the shelters. Shelters were asked to test between 50 and 200 dogs. Data were combined for shelters in neighboring counties with fewer than 50 samples. Finally, SNAP 4Dx Plus data were provided by investigators from two separate shelter studies: seven counties in Texas with data collected from March 2013 to December 2014 [13] and 10 counties in Mississippi with data collected from June 2016 up to and including February 2017 [12]. Additional file 1: Fig. S1 shows the geographic distribution of counties for which shelter data were included in this study; additional details can be found in Hazelrig et al. [14].

### Models

#### Owned dog population

A Bayesian spatial convolution model that includes random effects for both spatially correlated heterogeneity and uncorrelated heterogeneity [17–19] was used to estimate the prevalence in counties for which little to no data were reported during 2013 and up to and including 2019. The model is specified as follows:

$$Y_{ij} \,\sim \,Poisson\left( {p_{i} n_{ij} } \right)$$

1 $$log\left( {p_{i} } \right)\, = \,\beta_{0} + u_{i} + v_{i}$$

where ${Y}_{ij}$ is the count of positive tests, ${n}_{ij}$ is the total number of tests performed, and ${p}_{i}$ is the risk of testing positive in $i$ th county in the $j$ th year. Here, we assume that ${Y}_{ij}$ follows a Poisson distribution, a common choice for count data [19]. The linear predictor for $log({p}_{i})$ is comprised of a global intercept, ${\beta }_{0}$, a spatially uncorrelated random effect, ${v}_{i}$, and a spatially correlated random effect ${u}_{i}.$ To complete the Bayesian model, we specify normal priors for both ${v}_{i}$ and ${\beta }_{0}$ and an intrinsic conditional autoregressive prior for ${{\varvec{u}}=(u}_{1}...{u}_{M}){\prime};$ i.e., we specify that.

$$\beta_{0} \,\sim \,N\left( {0,\,\sigma_{{\beta_{0} }}^{2} } \right)$$

$$v_{i} \,\sim \,N\,\left( {0,\,\sigma_{v}^{2} } \right)$$

$$u_{i} |u_{j \ne i} \,\sim \,N\,\left( {\frac{{\mathop \sum \nolimits_{{j \in N_{i} }} u_{j} }}{{\left| {N_{i} } \right|}},\frac{{\sigma_{u}^{2} }}{{\left| {N_{i} } \right|}}} \right)$$

where ${N}_{i}$ is an index set that denotes the counties that share a border with the $i$ th county and $|{N}_{i}|$ denotes the number of such counties. The model was fit using integrated nested Laplace approximation (INLA) in R (Version 3.5.2 (2018–12-20)) using the package INLA [20]. Default prior and hyperprior settings were used, i.e., we specify

$$\tau_{v} \, = \,\frac{1}{{\sigma_{v}^{2} }}; log\left( {\tau_{v} } \right)\sim logGamma\left( {1,0.0005} \right)$$

$$\tau_{u} \, = \,\frac{1}{{\sigma_{u}^{2} }}; log\left( {\tau_{u} } \right)\sim logGamma\left( {1,0.0005} \right)$$

#### Association between owned dog seroprevalence and shelter-housed dog seroprevalence

To model the association between the two populations (owned and shelter-housed dogs), a binomial regression model was fit using the estimated owned dog seroprevalence from Eq. (1) as a predictor (after logit transformation) and the shelter-housed dog seroprevalence as the outcome. Only counties with data from the shelter-housed population were included in this model. The model specifications are as follows:

2 $$E\,\left( {q_{i} {|}\hat{p}_{i} } \right)\, = \,g^{ - 1} \,\left\{ {\alpha_{0} \, + \,\alpha_{1} \, \times \,\log \left( {\frac{{\hat{p}_{i} }}{{1\, - \,\hat{p}_{i} }}} \right)} \right\}$$

where $E\left(a|b\right)$ denotes the expected value of the random variable $a$ given the value of $b$, $g\left(\cdot \right)$ is the logit link, ${q}_{i}$ is the shelter-housed dog prevalence and ${\widehat{p}}_{i}$ is the estimated owned dog prevalence for the $i$ th county (i.e., data were matched by county), ${\alpha }_{0}$ is an intercept, and ${\alpha }_{1}$ is the slope parameter linking changes in owned and shelter-housed dogs. Model fitting was completed using the glm() function with a logit link in R. Fitted values, along with 95% confidence intervals, for the shelter-housed dog seroprevalence were obtained and plotted against the raw data. Note, if the confidence intervals did not capture the 1–1 line then we concluded that the shelter-housed dog seroprevalence was statistically different from the owned dog seroprevalence.

### Prediction of county-level shelter-housed dog seroprevalence

To predict the prevalence of the four pathogens in the shelter-housed dog population in counties not included in the study, we made use of the fitted models (Eq. 2) and the estimated owned dog prevalence (Eq. 1) for each county in the contiguous USA. Predictions for counties with an estimated owned dog prevalence outside the range considered in the development of the association models (i.e., Eq. 2) were excluded.

## Results

**Fig. 1:**
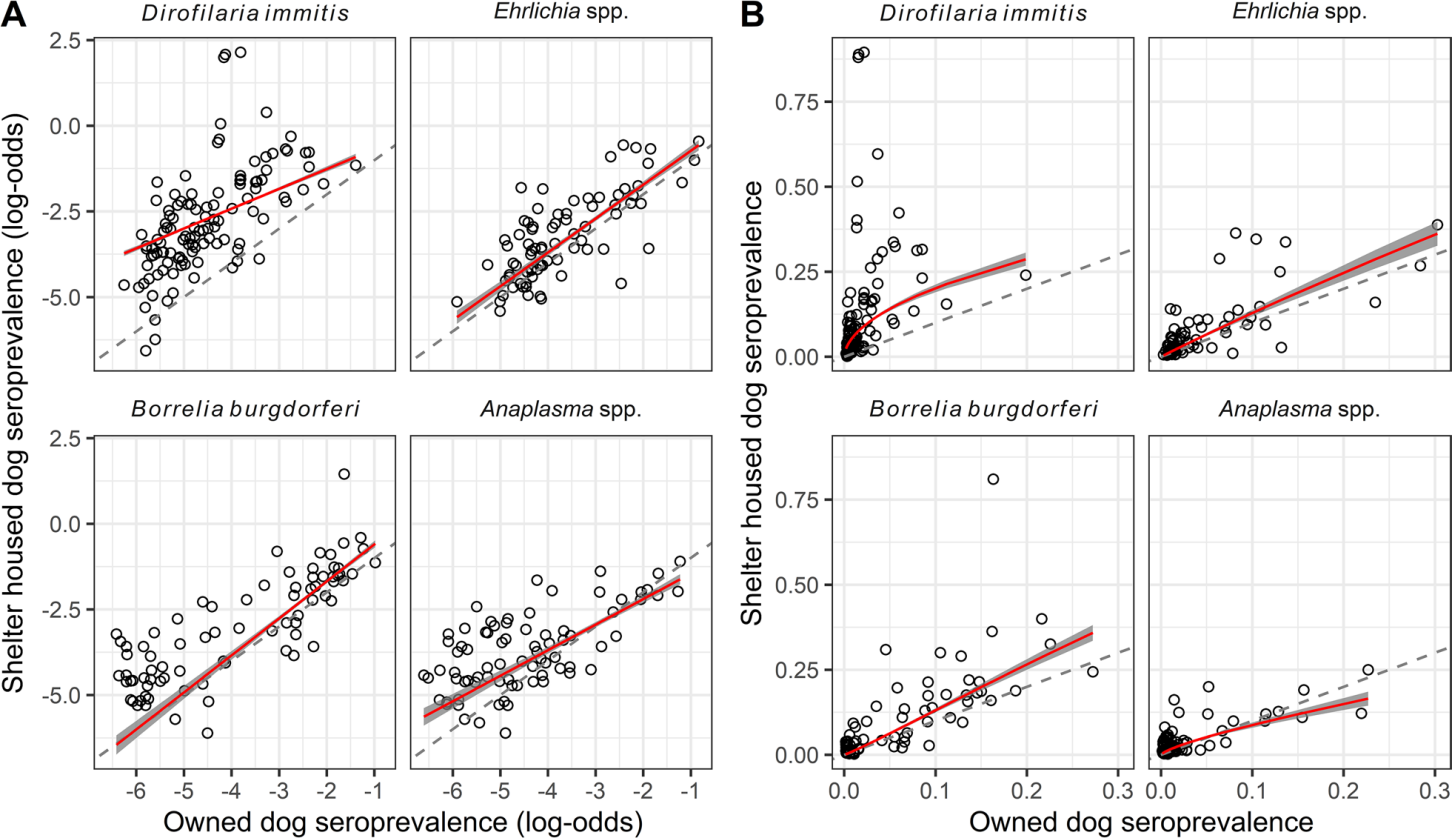
Association between the seroprevalence in the owned dog population (${p}_{i}$) and the shelter-housed dog population (${q}_{i}$) on the logit-transformed scale (**a**) or normal scale (**b**). Each point represents the log odds of the seroprevalence of a single shelter compared to the log odds of the seroprevalence of the owned population within the same county. The red line shows the fitted model (Eq. 2) that represents the association between the two populations for each pathogen. The thick grey line represents the 95% confidence interval for each model. The dashed line has a slope of 1 and indicates where the seroprevalence in the two populations is the same. Points above the line indicate a higher seroprevalence within the shelter-housed dog population, while points below the line indicate higher seroprevalence in the owned population

There was a positive linear association between the owned and high-risk populations for each of the four pathogens (Fig. 1a). To evaluate the seroprevalence on a normal scale, the model was plotted with the shelter and owned dog seroprevalence after the model fit was transformed (Fig. 1b). For *D. immitis*, *Ehrlichia* spp., and *B. burgdorferi*, the seroprevalence was higher among the shelter-housed dog population, with the gap between the populations increasing as seroprevalence increased (Fig. 1b). This was most marked for *D. immitis*. The seroprevalence of *Anaplasma* spp. was higher in the shelter population until around 6%, at which point that population had a lower seroprevalence compared to the owned population. To assess the estimates of the seroprevalence of the owned population obtained from the spatial convolution model (Additional file 2: Fig. S2), we plotted the model-based estimates of seroprevalence (Eq. 1) against the raw seroprevalence from the owned dog population to evaluate goodness of fit for the first model (Additional file 3: Fig. S3). These results indicate that the spatial convolution model fits the data well, with the primary discrepancies being for counties that report small numbers of tests (e.g., < 30 tests). These discrepancies are expected given that estimating seroprevalence based on a small number of tests is known to be problematic. Using a spatial model is a primary strength in the considered context, i.e., these models can leverage data from surrounding areas to provide more reliable assessments for areas where fewer data are available.

### Predicting county-level shelter-housed dog seroprevalence

**Fig. 2:**
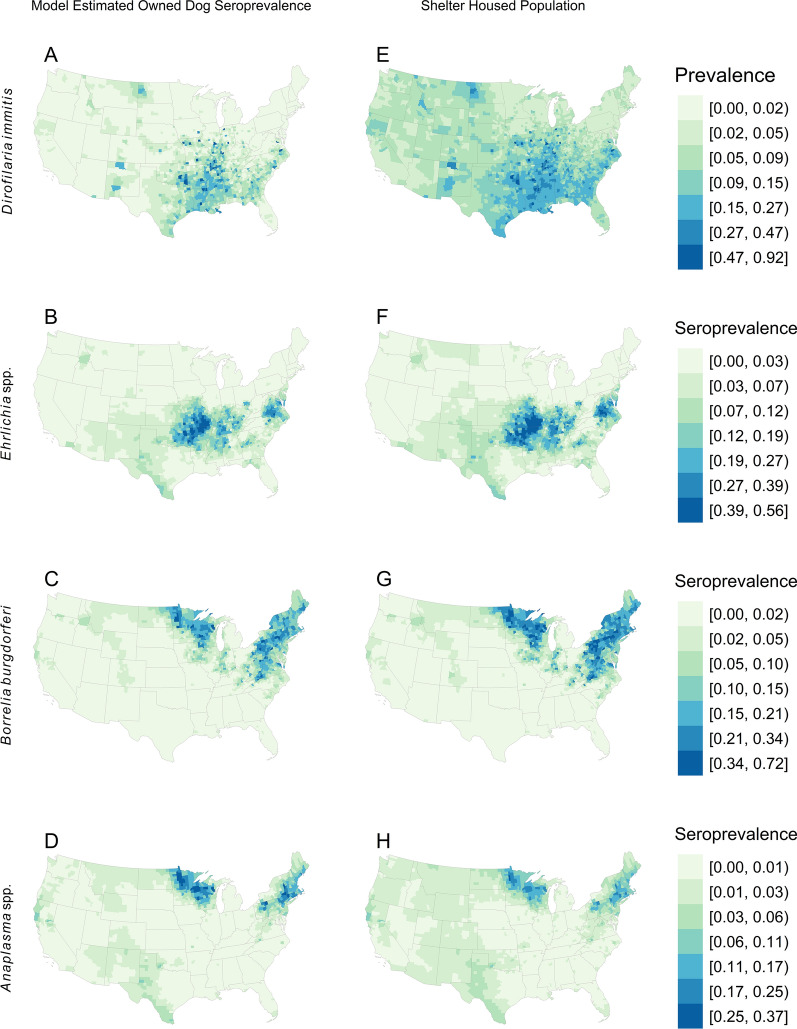
**a–h** Comparison of owned versus shelter-housed dog populations. Model estimated owned dog seroprevalence for four vector-borne pathogens [*Dirofilaria immitis* (**a**), *Ehrlichia* spp. (**b**), *B. burgdorferi* (**c**), and *Anaplasma* spp. (**d**)]. Model-predicted shelter-housed dog seroprevalence (Eq. 2) for the same four pathogens (**e**–**h**). Prevalence of each pathogen at the county level was predicted using the intercept and slope from each of the four regression models (Table 1) and the estimated owned dog seroprevalence, ${p}_{i}$

We predicted the seroprevalence of each pathogen at the county level by using the estimated intercept and slope from each of the four regression models (one for each pathogen; Table 1) and the estimated owned dog seroprevalence (Fig. 2a–d). The predicted shelter-housed dog seroprevalences, based on Eq. (2), for each of the four pathogens are shown in Fig. 2e–h.

**Table 1:** Regression coefficients

Pathogen	Value	Estimate	SE	*P*-value
*Dirofilaria immitis*	Intercept	−0.105	0.07	0.137
	Coefficient	0.579	0.017	0
*Ehrlichia* spp.	Intercept	0.257	0.101	0.011
	Coefficient	0.989	0.031	0
*Anaplasma* spp.	Intercept	−0.707	0.115	0
	Coefficient	0.746	0.035	0
*Borrelia burgdorferi*	Intercept	0.48	0.081	0
	Coefficient	1.08	0.034	0

Overall, the findings from this study suggest that the prevalence of heartworm infection and seroprevalence of *Ehrlichia* spp. and *B. burgdorferi* are higher in shelter-housed dogs across the USA, regardless of their location, compared with those in the owned population. The seroprevalence of *Anaplasma* spp. is predicted to be higher in areas that generally have very low to low seroprevalence, but unexpectedly, in areas of higher seroprevalence within the owned population, the seroprevalence is expected to be lower in the shelter-housed dog population.

## Discussion

Underestimating the risk of vector-borne pathogen exposures could impact a decision to implement preventative care measures (such as heartworm chemoprophylaxis or use of products that repel or kill fleas, ticks, or mosquitoes) that reduce canine infection and disease. The decision to test for diagnostic or screening purposes is often based on the perceived risk of exposure to a particular pathogen, and it may be inappropriate to base this decision on the risk to another population. For example, not testing dogs in a higher risk population (e.g., strays) based on information and seroprevalence estimated from dogs receiving routine preventative care would lead to many missed diagnoses. Finally, animal shelters often have limited resources and must prioritize the testing and treatments that they perform. Uncertainty in the knowledge of the risk of exposure in non-owned dogs makes it difficult for shelters to make informed decisions as to whether testing or screening is appropriate for their population.

County-level maps which are updated monthly and show the distribution and prevalence of four important vector-borne pathogens of dogs are an important resource and are available from CAPC (www.capcvet.org). This mapping effort has been ongoing in all 50 states since 2012, and has been recently expanded to include Canada. These data have allowed for descriptive studies on the prevalence of exposure [1, 2], analysis of the temporal trends in prevalence [21–25], predictive models that forecast the expected prevalence for the upcoming year [26–30], and studies that examine the relationship between Lyme disease in humans and canine exposure to *B. burgdorferi* [31]. However, common discussions regarding these data and studies revolve around the assumption that the dogs are likely in the care of a veterinarian, owned, and provided some protection for exposure to fleas, ticks, and mosquitoes. The American Society for the Prevention of Cruelty to Animals estimates that 3.3 million dogs enter a shelter annually [32]. Although this number is declining, this is a large population of animals that is not well represented by the aforementioned studies or current prevalence estimates [4]. Through county-level comparisons, the present study does show that, in general, CAPC data underestimate the actual prevalence of vector-borne pathogen exposures in shelter-housed dogs. Thus, decisions made about testing or screening for vector-borne pathogens based on current estimates, especially for heartworm, are made using data that underestimate the actual prevalence.

Not surprisingly, we found that shelter-housed dogs were significantly more likely to test positive for heartworm compared to their owned counterparts in the same geographical location, even in areas of low prevalence (based on owned dog data). This is supported by a Florida study [9] that found that heartworm prevalence in shelter dogs was over 10% higher than in owned dogs (1.4% vs 14.6%). This was true for most tick-borne pathogens as well, with shelter-housed dogs significantly more likely to test positive in areas of very low to low seroprevalence (based on owned dog data). A surprising finding in this study was that in some high prevalence areas of *Anaplasma* spp. the owned dog population frequently had a higher seroprevalence. Several factors could be related to this finding. First, the average age of dogs entering a shelter is likely to be lower than that of the owned population. In the study by Tzipory et al. [9] comparing prevalences of vector-borne infections, the mean age of pet dogs was ~ 8 years compared to 2.5 years for shelter-housed dogs. A relatively low average age of shelter animals was also observed in a study on heartworm prevalence in shelter dogs in Mississippi [12], where 73.5% of dogs were under the age of 3 years. Dogs exposed to and not treated for tick-borne infections may have antibodies that persist for years. Mircean et al. [33] found the seroprevalence for *Anaplasma* spp. and *E. canis* to be significantly higher in dogs that received prophylactic treatment than in those that did not, and dogs over the age of 2 years had a higher prevalence of both pathogens when compared to dogs under 2 years [33]. Because of repeated exposure when prophylactic treatments had not been used, older dogs may have had more opportunity for exposure and therefore a higher probability of having been exposed during their lifetime. However, our finding should be interpreted with caution because the association seen in this study could be due to other factors (e.g., urban vs rural dogs). Future studies aimed at understanding these factors are needed to better understand why the owned dogs in this study were more likely to test positive in some areas.

Although the current study includes shelters from many locations throughout the contiguous USA, there were not enough data to fully account for all of the spatially related covariates. When we predict the shelter-housed dog seroprevalence in areas outside regions for which we have data, we are assuming that there is little to no impact of various unmeasured covariates. Specifically, the three major reasons for a dog to enter a shelter are owner relinquishment, or that it is a stray, or that it is a transfer from another organization [34]. We eliminated most transfers by including only dogs that were relinquished or picked up within that county or neighboring counties. However, reasons for owner relinquishment and for a dog to be unwanted/a stray vary across different regions of the USA [34]. They include socioeconomic factors, differing views on pet ownership, and how dog populations are managed (e.g., breeding and spay/neuter programs). Shelters should interpret these results within the context of what they know about their own population of dogs. Finally, despite this study focusing on shelter-housed dogs as a proxy for a high-risk population of dogs, owner-relinquished dogs may have also received veterinary care. Thus, the best representation of natural transmission and risk would be to sample dogs that are known to have not received any care; for heartworm, sampling of wild canids could also be useful. However, access to these populations (e.g., owned dogs that receive no care) is difficult and sampling these dogs could come with ethical considerations (e.g., when testing a dog whose owner cannot afford preventatives or treatment, should researchers offer treatment if the dog tests positive for heartworm?).

This study did not include a temporal component. There is evidence that the seroprevalences of the pathogens examined in this study are changing over time in the owned group [23–25]. The assumption in the present study was that the association between these two groups would not change over time; however, this needs to be investigated in future work. As the geographic distribution of vectors and their pathogens shift, the transmission pressure on these two dog populations may differ from what is currently occurring at the sites included in our study. In addition, novel tick species or pathogens may be introduced into new regions in which they may have different transmission dynamics (e.g., [35–37]).

## Conclusions

This study showed that there is an association between prevalences of selected vector-borne pathogens in shelter-housed and owned dog populations. This association was used to create maps of predicted shelter-housed dog seroprevalence, which showed that the expected prevalence in dogs entering shelters is higher in many regions of the USA. If shelters and veterinarians are making the decision to not screen these dogs based on the known seroprevalence of the owned group, they are likely underestimating the risk of exposure. This is especially true for heartworm. Most shelters (~ 70%) in high prevalence areas of canine heartworm do test [5], but across the USA, only ~ 50% of shelters test [38]. By providing an estimate of the seroprevalence in shelter-housed dogs throughout the USA, shelters and veterinarians can make informed evidence-based decisions on whether testing and screening for these pathogens is appropriate for their local dog population. While advocating for additional testing of shelter-housed dogs is an important step forward and generates valuable data, even these data are not representative of the actual risk of vector-borne pathogen infections of dogs. To establish a more accurate representation, future work needs to test populations of stray and shelter-housed dogs that are known to have had no preventative care, or owned dogs with no history of preventative treatment. However, our work represents an important step in understanding the relationships in the seroprevalences of vector-borne pathogens between groups of high-risk and owned dogs, and provides valuable data on the risk of vector-borne diseases in dogs.

### Supplementary Information

**Additional file 1: Figure S1.** Map showing the location of the counties containing a shelter included in this study. This includes all the data sources mentioned in the text: current study, Hodo et al. [13], Donnett et al. [12], and Idexx Laboratories. Data from counties in grey were aggregated with those of neighboring counties in red due to small sample sizes.

**Additional file 2: Figure S2.** Maps of raw, owned dog seroprevalences by county for four vector-borne pathogens [*Dirofilaria immitis* (**A**); *Ehrlichia* spp. (**B**), *Borrelia burgdorferi* (**C**); and *Anaplasma* spp. (**D**)]. Model estimated prevalences of the owned dog population (Eq. 1) for the same four pathogens (**E**–**H**).

**Additional file 3: Figure S3**. Comparison of the raw owned dog seroprevalences and model estimated seroprevalences of the owned dog population (Eq. 1) by county. Sample sizes for the owned dog population ranged from 1 to hundreds of thousands of tests for the study period. The dot size corresponds to the number of tests. Counties with more than 30 tests (the vast majority) fall on a straight line, indicating that the estimated values closely align with the raw prevalences. Those in blue are counties with fewer than 30 tests**.** Deriving estimates of county level prevalence from our spatial model overcomes the unreliability of prevalences from these counties by borrowing information from surrounding counties to help guide the estimates.

## Data Availability

The owned dog population dataset analyzed during the current study is available from http://www.capcvet.org. Aggregated data from shelters are available from the corresponding author on reasonable request.
